# Down‐regulated in OA cartilage, SFMBT2 contributes to NF‐κB‐mediated ECM degradation

**DOI:** 10.1111/jcmm.13826

**Published:** 2018-08-22

**Authors:** Safdar Hussain, Mengyao Sun, Zixin Min, Yuanxu Guo, Jing Xu, Nosheen Mushtaq, Lisong Heng, Huang Huang, Yitong Zhao, Ying Yuan, Nazim Hussain, Fujun Zhang, Yan Han, Peng Xu, Jian Sun, Shemin Lu

**Affiliations:** ^1^ Department of Biochemistry and Molecular Biology School of Basic Medical Sciences Health Science Center Xi'an Jiaotong University Xi'an China; ^2^ Centre for Applied Molecular Biology (CAMB) University of the Punjab Lahore Pakistan; ^3^ Department of Microbiology School of Basic Medical Sciences Health Science Center Xi'an Jiaotong University Xi'an China; ^4^ Department of Orthopedics and Traumatology Honghui Hospital Health Science Center Xi'an Jiaotong University Xi'an China; ^5^ Key Laboratory of Environment and Genes Related to Diseases Ministry of Education Xi'an China

**Keywords:** SFMBT2, NF‐κB, ECM metabolism, cartilage, OA

## Abstract

The interplay between anabolic and catabolic factors regulates cartilage matrix homoeostasis. In OA, this balance is disrupted which results in cartilage degradation involving a plethora of inflammatory factors. Here, we identify a novel gene “Scm‐like with four MBT domains protein 2” (SFMBT2) negatively regulated in OA cartilage. Articular cartilage from human OA patients undergoing knee arthroplasty surgery exhibited significantly decreased levels of SFMBT2 compared to the normal controls. Down‐regulation of SFMBT2 by specific siRNA disturbed the metabolic homoeostasis and led to decreased expression of anabolic genes (SOX9, COL2A1) while increasing the expression of catabolic genes (MMP13 and ADAMTS4), in human chondrocytes. Finally, we revealed that SFMBT2 intervention by siRNA contributed to the catabolic phenotype of human chondrocytes mediated by NF‐kB pathway.

## 1 INTRODUCTION

Osteoarthritis (OA) is a painful chronic inflammation of the joint associated with cartilage degeneration. It is the most prevalent form of arthritis affecting millions of persons worldwide, imposing a considerable socio‐economic burden.^1^ Despite the multifactorial nature of its aetiology, the experimental data provided in many of the studies suggest that OA is linked with alterations of the genes expressions rather than modification in the genetic code itself.^2^ Reports on chondrocytes behaviour in OA suggest that at different stages and/or locations within articular cartilage, extracellular matrix (ECM) metabolism is regulated through coordinated mechanisms that are not fully understood. In this study we demonstrate down‐regulation of SFMBT2 in OA contributes to ECM degradation via activation of the NF‐κB signalling pathway.

## 2 MATERIALS AND METHODS

Cartilage samples were collected from 10 human OA patients (diagnosed with Kellgren and Lawrence grade IV osteoarthritis), undergoing knee arthroplasty surgery (6 women and 4 men; age range 45‐69 years). Normal cartilage specimens (controls) were obtained from five donors (2 women and 3 men; age range 38‐66 years) at autopsy. All the samples were collected from Honghui Hospital, Xi'an, Shaanxi, China. Accordingly, the study was performed with approval of the Ethical Committee of Xi'an Jiaotong University, Health Science Center, and all individuals provided full written informed consent.

In this study, we employed C28/I2 and SW1353 human chondrocytes and transfected them with specific small‐interfering RNA (siRNA) sequence against SFMBT2 (si‐*SFMBT2*) or a scrambled (negative control) siRNA (si‐NC) sequence (Table S1), with Lipofectamine™ 2000 (Thermo Fisher Scientific, Shanghai, China). TNF‐α (Sigma, Shanghai, China) and IL‐1β (Sigma, Shanghai, China; see Figure S2) were used to induce inflammation in the human chondrocytes, and BAY 11‐7082 (Abcam, Shanghai, China) was used to block the NF‐κB signals (specific details are depicted in the respective figure legends).

All the molecular expressions at mRNA level were determined by RT‐qPCR with the specific primers (Table S2) and at protein level by Western blotting (WB) & Immunohistochemistry (IHC) with respective antibodies (Table S3).

## 3 RESULTS AND DISCUSSION

### 3.1 SFMBT2 is down‐regulated in OA cartilage

SFMBT2 is a member of polycomb group (PcG) of proteins and is known to repress HOXB13 gene expression via its association with methylated histones H3 and H4.^3^ However, no study stating its role in OA/cartilage has yet been reported. In a previous study at our department, differentially expressed genes (DEGs) in OA cartilage were screened by suppression subtractive hybridization (SSH). SFMBT2 was identified from the reverse subtraction library by sequence analysis and a similarity search with the BLAST programme.^4^ We selected this gene for further investigations in this study.

**Figure 1:**
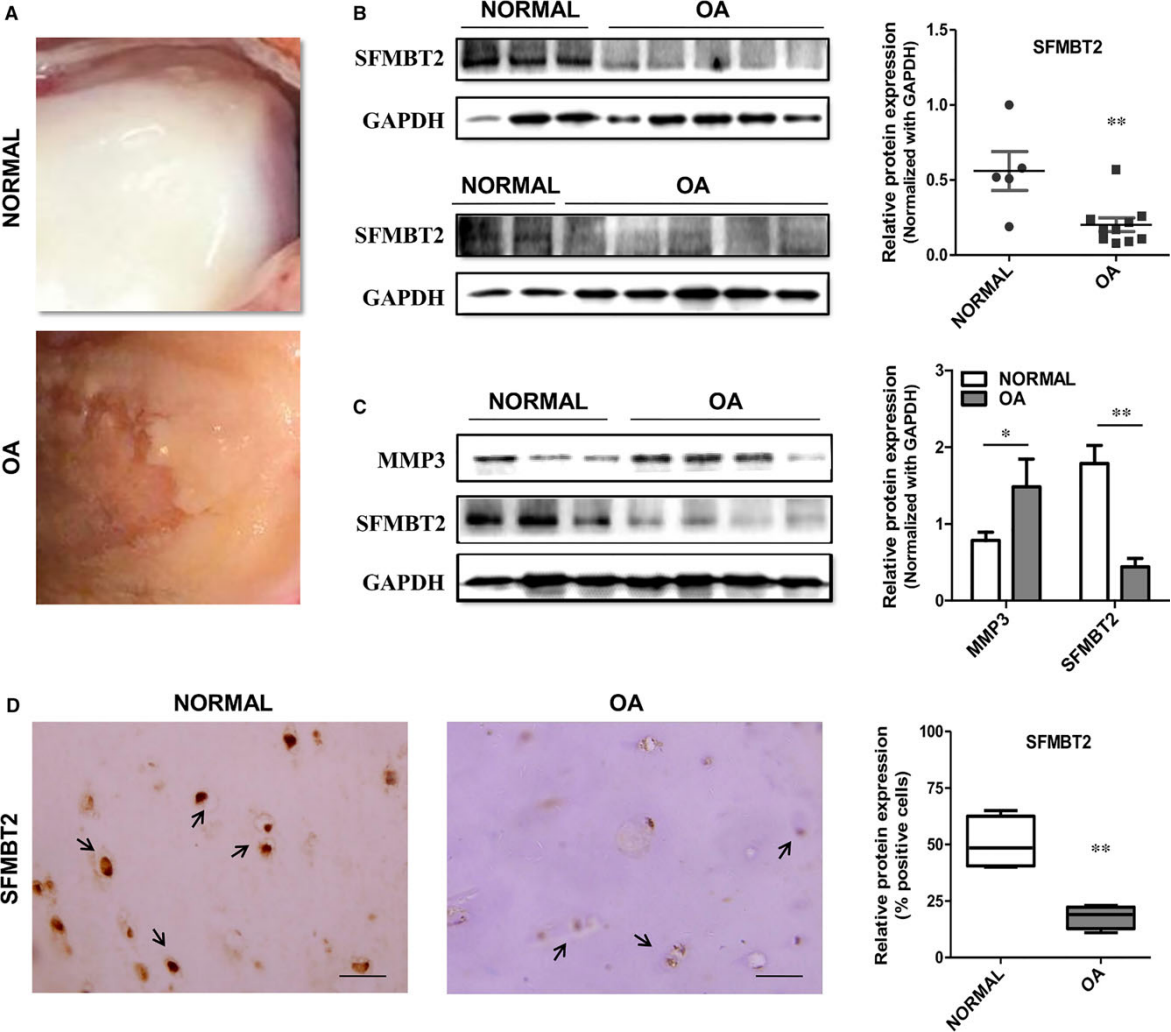
Expression of SFMBT2 in ‘OA vs Normal’ Cartilage: A, Representative macromorphological pictures of the human knee ‘NORMAL’ articular cartilage (upper panel) and ‘OA’ cartilage (lower panel). B, Protein expression of SFMBT2 in the cartilage from ten human ‘OA’ patients and five ‘NORMAL’ controls, determined by Western blotting (left panel) and normalized with the house‐keeping gene GAPDH (right panel). C, Comparative analysis of SFMBT2 and MMP3 expression determined by Western blotting (left panel) and normalized with GAPDH (right panel) in articular cartilage from the four human ‘OA’ patients and three ‘NORMAL’ controls. D, Representative IHC staining images of SFMBT2 expression in ‘OA’ patients’ vs ‘Normal’ cartilage samples from the controls. Arrows indicate SFMBT2‐positive cells (left panel). Statistical analysis of per cent positive cells for SFMBT2 in IHC detection from OA vs Normal cartilages (right panel). Scale bar, 100 μm. Student's *t* test was used to identify the differences between two groups. * and ** stand for *P*‐values less than 0.05 and 0.01 respectively

Western blot analysis of the ‘OA’ cartilage samples from human patients displayed significantly decreased levels of SFMBT2 (*P* = 0.003) when compared with ‘Normal’ cartilages from the controls **(**Figure 1B). In contrast, protein expression of catabolic marker gene MMP3, which belongs to the family of matrix metalloproteinases (MMPs), was up‐regulated radically (*P* = 0.026) in ‘OA’ cartilage samples (Figure 1C). IHC staining of the cartilage specimens exhibited a significantly decreased expression of SFMBT2 (*P* = 0.0023) in ‘OA’ patients as compared with the ‘Normal’ controls (Figure 1D). These data establish a negative association of SFMBT2 with OA, however, mechanism behind the down‐regulation of this gene in OA needs further exploration.

### 3.2 SFMBT2 knockdown dysregulates the metabolic homeostasis of chondrocytes

**Figure 2:**
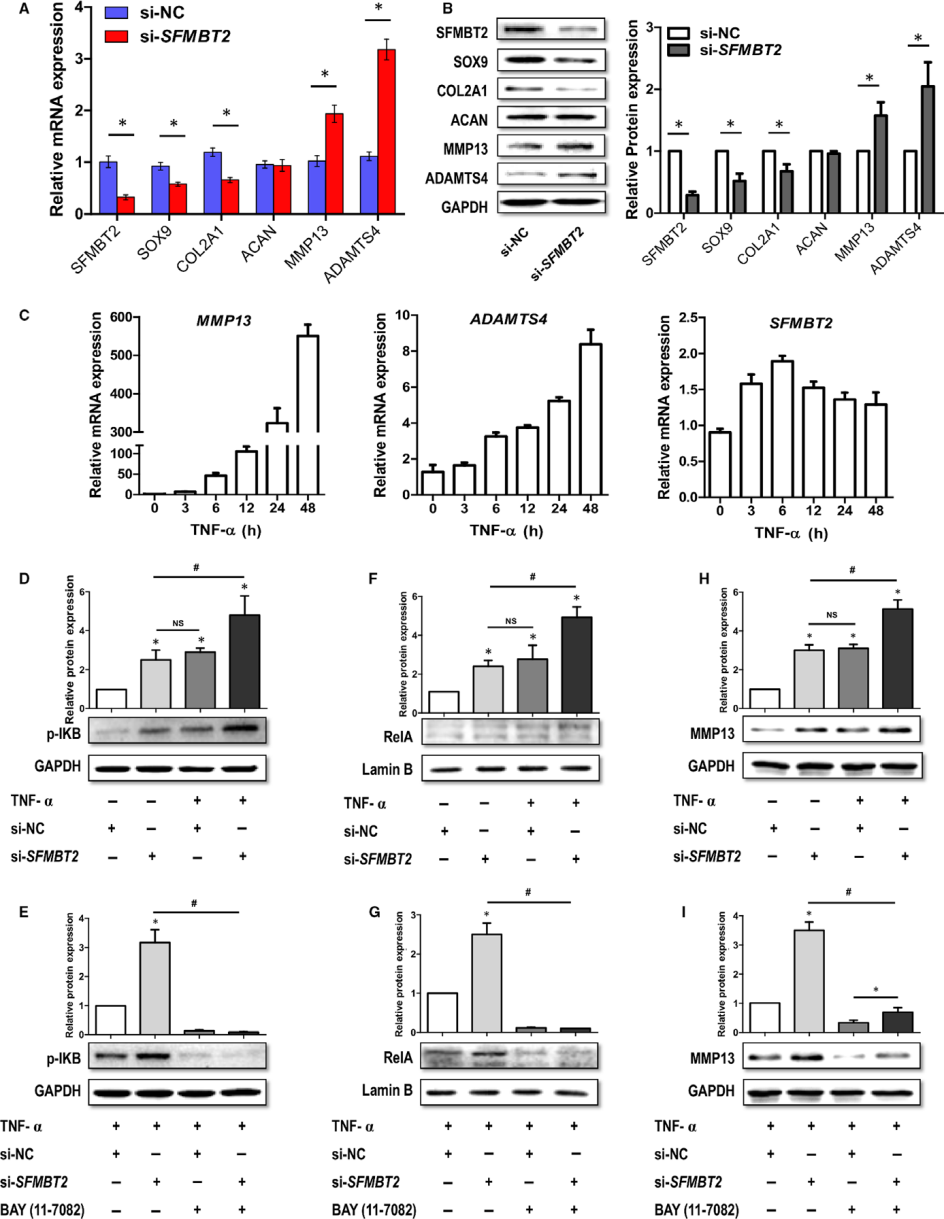
Impact of SFMBT2 intervention by siRNA in human chondrocytes (C28/I2 and SW1353) and the downstream regulatory mechanism of ECM degradation by si‐*SFMBT2* mediated by NF‐κB: A&B, C28/I2 cells transfected with 80 nmol/L of SFMBT2‐specific siRNA (si‐*SFMBT2*) or scrambled (negative control) siRNA (si‐NC) and the effect of SFMBT2 knockdown on the expression of key chondrogenic genes (SOX9, COL2A1, ACAN, MMP13 and ADAMT4) measured at mRNA and protein levels, determined by RT‐qPCR (A) and Western blotting (B) respectively. C, SW1353 cells stimulated with TNF‐α (10 ng/mL) for different time points and the mRNA levels of SFMBT2, MMP13 and ADAMTS4 determined by RT‐qPCR. D, WB detection of IκB phosphorylation signals in SW1353 cells transfected with si‐NC or si‐*SFMBT2* (80 nmol/L) and stimulated with or without TNF‐α (10 ng/mL). *: compared with si‐NC group; #: compared with both si‐*SFMBT2* and TNF‐α+si‐NC groups. E, Detection of IκB phosphorylation signals in SW1353 cells transfected with si‐NC or si‐*SFMBT2*, stimulated with TNF‐α and incubated with or without NF‐κB inhibitor (BAY 11‐7082, 10 μmol/L/mL). *: compared with TNF‐α+si‐NC group; #: compared with both TNF‐α+si‐NC+inhibitor and TNF‐α+si‐*SFMBT2* + inhibitor groups (F) Detection of RelA in the nuclear fraction of SW1353 cells transfected with si‐NC or si‐*SFMBT2* and stimulated with or without TNF‐α. *: compared with si‐NC group; #: compared with both si‐*SFMBT2* and TNF‐α+si‐NC groups. G, Detection of RelA in the nuclear fraction of SW1353 cells transfected with si‐NC or si‐*SFMBT2,* stimulated with TNF‐α and incubated with or without NF‐κB inhibitor. *: compared with si‐NC group; #: compared with both TNF‐α+si‐NC+inhibitor and TNF‐α+si‐*SFMBT2* + inhibitor groups. H, Analysis of MMP13 levels in SW1353 cells transfected with si‐NC or si‐*SFMBT2,* and treated with or without TNF‐α. *: compared with si‐NC group; #: compared with both si‐*SFMBT2* and TNF‐α+si‐NC groups. I, Detection of MMP13 levels in SW1353 cells transfected with si‐NC or si‐*SFMBT2,* stimulated with TNF‐α and incubated with or without NF‐κB inhibitor. *: compared with TNF‐α+si‐NC group; #: compared with both TNF‐α+si‐NC+inhibitor and TNF‐α+si‐*SFMBT2* + inhibitor groups. GAPDH was used as internal control in RT‐qPCR and Western blot detections. Protein (WB) bands were quantitatively analysed by Image J software. All data represent mean ± SEM. Non‐parametric Mann‐Whitney *U* test was used to determine the differences between two groups. Both ‘*’ and ‘#’ stand for *P*‐value ≤ 0.05. Two different experimental approaches were followed to detect the downstream signals of the TNF‐α stimulation, and the downstream genes transcribed by NF‐κB, ie, (a) In signalling experiments, SW1353 cells were transfected with SFMBT2 siRNA along with the negative control siRNA for 24 h, subjected to BAY 11‐7082 for 30 min and then treated with or without TNF‐α for 1 h (C28/I2 for 30 min, data not shown); (b) To detect the expression of downstream target genes of NF‐κB (MMPs, ADAMTSs), SW1353 cells were transfected with SFMBT2 siRNA along with the negative control siRNA for 36 h, subjected to BAY 11‐7082 for 2 h (C28/12 for 1 h, data not shown) and then treated with or without TNF‐ α for 12 h

Three different siRNA sequences targeting SFMBT2 mRNA (Table S1) were synthesized and tested to intervene its expression in C28/I2 cells (Figure S1). Endogenous SFMBT2 was knocked down >70% by 80 nmol/L of the effective siRNA sequence (siR3). SFMBT2 interference altered the expression of key metabolic genes in C28/I2 chondrocytes. SOX9 and COL2A1 were decreased, whereas MMP13 and ADAMTS4 were increased significantly (*P* < 0.05) by si‐*SFMBT2*, both at mRNA and protein levels, determined by RT‐qPCR (Figure 2A) and Western blotting (Figure 2B) respectively. Expression of Aggrecan (ACAN) did not change indicating no immediate effect of si‐*SFMBT2* on it. SOX9 is a pivotal transcriptional regulator and is essential for chondrocytes phenotypic stability, differentiation^5^ and proliferation.^6^ Down‐regulation of SOX9 may induce angiogenesis, cartilage resorption and formation of bone marrow and endochondral bone trabeculae,^7^ which are associated with OA progression. MMPs and ADAMTSs are known to accelerate the catabolic process and are involved in ECM degradation within the cartilage.^8^, ^9^ Our results indicate that certain levels of SFMBT2 are imperative to maintain the normal metabolic homeostasis, and its down‐regulation may promote catabolic phenotype of the chondrocytes.

### 3.3 SFMBT2 intervention by siRNA contributes to NF‐κB‐mediated ECM degradation

Pro‐inflammatory cytokines such as TNF‐α and IL‐1β can sponsor catabolism and enhance the expression of matrix degrading genes.^10^ TNF‐α triggers the catabolic process by activating downstream signalling pathways such as Nuclear Factor kappa B (NF‐κB) and Mitogen‐Activated Protein Kinases (MAPKs).^11^, ^12^ Active NF‐κB is involved in the regulation of various target genes, including chemokines, transcription factors, growth factors, enzymes and cell‐cycle regulators, immune receptors, regulators of apoptosis, stress response genes and adhesion molecules.^11^, ^13^

To depict the mechanism behind the up‐regulation of catabolic genes (MMP13 and ADAMTS4) by si‐*SFMBT2*, we treated SW1353 cells with TNF‐α and detected the downstream inflammatory signals of NF‐κB pathway. TNF‐α treatment led to the increased expression of *MMP13* and *ADAMTS4* in a time‐dependant manner, determined by RT‐qPCR (Figure 2C). Expression of *SFMBT2* initially increased (6 hours), but then displayed a gradually decreasing trend (12‐48 hours) under the TNF‐α treatment.

Next, SW1353 cells were transfected with si‐NC or si‐*SFMBT2* and stimulated with or without TNF‐α in the presence or absence of the specific NF‐κB inhibitor (BAY 11‐7082). Normally, NF‐κB dimers are maintained in the cytosol of unstimulated cells, complexed with IκB proteins.^14^ Upon activation by a large number of stimuli (including TNF‐α), the IκB proteins undergo phosphorylation, ubiquitylation and proteasome‐mediated degradation, which results in the liberation of NF‐κB dimers followed by their nuclear translocation.^15^ As shown in Figure 2D, TNF‐α treatment led to the increased phosphorylation of IκB, accompanied by the activation of NF‐κBp65 (RelA) (Figure 2F). Interestingly, si‐*SFMBT2* mimicked the act of TNF‐α in the unstimulated chondrocytes. In addition, si‐*SFMBT2* boosted up the signals of IκB phosphorylation, when used in combination with TNF‐α, and subsequently increased the levels of RelA (Figure 2D,F). However, BAY 11‐7082 inhibited the IκB phosphorylation and subsequent levels of RelA, both in TNF‐α+si‐NC‐ and TNF‐α+si‐*SFMBT2‐*treated chondrocytes (Figure 2E,G).

We further detected the expression of MMP13, a target gene of RelA, and observed that TNF‐α stimulation led to the increased expression of MMP13 in SW1353 cells. As expected, si‐*SFMBT2* chondrocytes also exhibited the increased levels of MMP13, treated with or without TNF‐α (Figure 2H). BAY11‐7082 reduced the transcription of MMP13; however, the reduction was less in si‐*SFMBT2* group as compared with the si‐NC group (Figure 2I). This might be as a result of the other inflammatory factors regulating the MMP13 expression, associated with si‐*SFMBT2* which needs further explorations.

## CONFLICT OF INTERESTS

The authors have no conflict of interest to declare.

## AUTHOR'S CONTRIBUTIONS

S.H. designed and executed the experiments, analysed the data and wrote the manuscript. M.S., Y.G. and J.X. helped perform the analysis with constructive discussions. N.M., H.H., Y.Z., Y.Y., N.H., F.Z. and Y.H. helped the experiments. P.X. and L.H. provided the OA and control samples. S.L. and J.S. supervised the study and critically revised the manuscript for important intellectual content.
