# Supplementary material for: Down‐regulated in OA cartilage, SFMBT2 contributes to NF‐κB‐mediated ECM degradation
Source: J Cell Mol Med. 2018 Aug 22;22(11):5753–8. doi: 10.1111/jcmm.13826 (PMC6201222; doi:10.1111/jcmm.13826)
Supplement: Supplementary file 1 [file JCMM-22-5753-s001.docx]

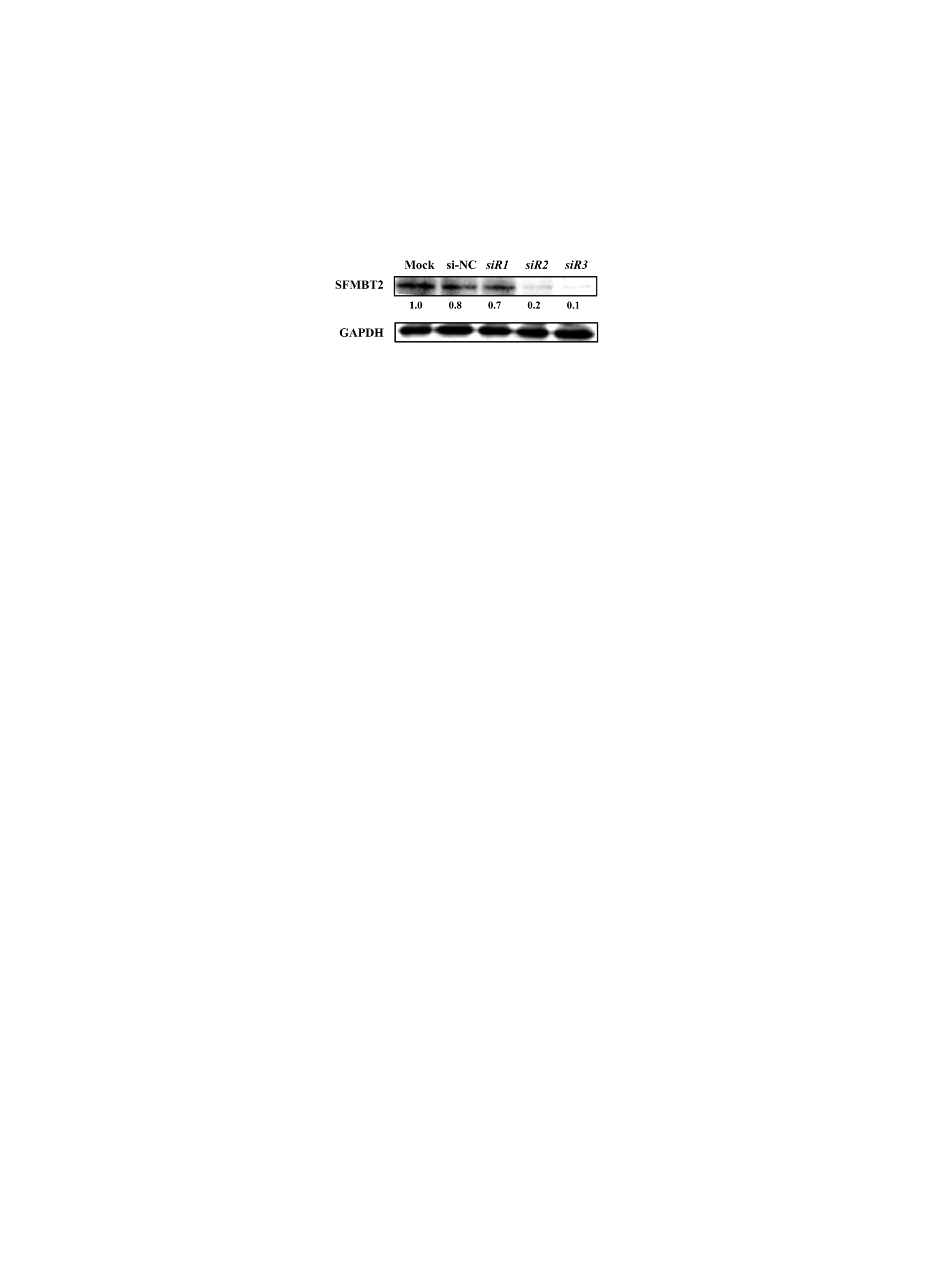


**Figure S1: SFMBT2 siRNA optimization**

Three different siRNA sequences against SFMBT2 (siR1, siR2, siR3) along with a scrambled siRNA (negative control) sequence, were used to transfect C28/I2 cells with lipofectamine 2000. The cells were harvested 48 h after the transfection. Protein expression of SFMBT2 was determined by Western blotting and normalized with GAPDH. *siR3* was found the most effective siRNA sequence and was used in subsequent SFMBT2 knock down experiments.

**Figure S2:** **Treatment of SW1353 cells with IL-1β**

SW1353 cells were treated with 10 ng/mL of IL-1β for different time-points. The mRNA levels of *MMP13* and *SFMBT2* were determined by RT-qPCR. GAPDH was used as the internal control.

**Table S1: Oligonucleotides siRNA Sequences**

| Gene Name | siRNA Oligonucleotides Sequence (5’ – 3’) |
| --- | --- |
| hsa-SFMBT2 siRNA1 (sense) | GCUUCCGAAAUACAUCAUUTT |
| hsa-SFMBT2 siRNA1 (anti-sense) | AAUGAUGUAUUUCGGAAGCTT |
| hsa-SFMBT2 siRNA2 (sense) | GCCUCUGAAUGGAAAUGUATT |
| hsa-SFMBT2 siRNA2 (anti-sense) | UACAUUUCCAUUCAGAGGCTT |
| hsa-SFMBT2 siRNA3 (sense) | GCACUUUGUCAGCUUCCAATT |
| hsa-SFMBT2 siRNA3 (anti-sense) | UUGGAAGCUGACAAAGUGCTT |
| siRNA Negative Control (sense) | UUCUCCGAACGUGUCACGUTT |
| siRNA Negative Control (anti-sense) | ACGUGACACGUUCGGAGAATT |

**Table S2: messenger RNA (mRNA) specific primers used in RT-qPCR**

| **Gene** | **Sequence (5’ – 3’)** | **Ta (^o^C)** |
| --- | --- | --- |
| hsa-SFMBT2 | **F:** ACGAAACAGGAGGAGGAGGAGAG | 64 |
|  | **R:** GGAAGGGTCAGAAGCAGGAGTG |  |
| hsa-SOX9 | **F:** CGCACATCAAGACGGAGCAG | 61 |
|  | **R:** TGTAGGTGAAGGTGGAGTAGAGG |  |
| hsa-ACAN | **F:** ATGCCCAAGACTACCAGTGG | 61 |
|  | **R:** TCCTGGAAGCTCTTCTCAGT |  |
| hsa-COL2A1 | **F:** TACCACTGCAAGAACAGC | 61 |
|  | **R:** GTGCAATGTCAATGATGG |  |
| hsa-MMP3 | **F:** CTGGACTCCGACACTCTGGA | 61 |
|  | **R:** CAGGAAAGGTTCTGAAGTGACC |  |
| hsa-MMP13 | **F:** AATATCTGAACTGGGTCTTCCAAAA | 61 |
|  | **R:** CAGACCTGGTTTCCTGAGAACAG |  |
| hsa-ADAMTS4 | **F:** CGCACCGACCTCTTCAAG | 59 |
|  | **R:** CTCCAGCACATAGTAGTAGCC |  |
| hsa-ADAMTS5 | **F:** GCTGTGCTGTGATTGAAGAC | 59 |
|  | **R:** ATGCTGGTAAGGATGGAAGAC |  |
| hsa-GAPDH | **F:** CACCCACTCCTCCACCTTTG | 61 |
|  | **R:** CCACCACCCTGTTGCTGTAG |  |

**Table S3 Antibodies used in the study**

| Antibody | Cat # | Company | | Application / dilution |
| --- | --- | --- | --- | --- |
| SFMBT2 | 25256-1-AP | Protein tech. China | WB (1:500) | |
| SFMBT2 | bs-21105R | Bioss, China | IHC (1:50) | |
| SOX9 | Ab182579 | Abcam, China | WB (1:1000) | |
| Aggrecan | 13880-1-AP | Protein Tech. China | WB (1:500) | |
| COL2A1 | BA0533 | BOSTER, China | WB (1:200) | |
| MMP3 | bs-0413R | Bioss, USA | WB (1:400) | |
| MMP13 | MAB511 | R & D Systems | WB (1:400) | |
| ADAMTS4 | A2525 | Abclonal, USA | WB (1:500) | |
| NF-kBp65 | 8242 | CST, USA | WB (1:200) | |
| p-IKB (S32) | Ab92700 | Abcam, China | WB (1:1000) | |
| GAPDH | 10494-1-AP | Protein tech, China | WB (1:2000) | |
| Lamin B | 66095-1-Ig | Protein tech, China | WB (1:1000) | |
| HRP conjugated anti-  Rabbit IgG (2ndary Ab) | 31460 | Thermo Fisher, USA | WB (1:2000) | |
